# Feasibility of using a depth camera or pressure mat for visual feedback balance training with functional electrical stimulation

**DOI:** 10.1186/s12938-023-01191-y

**Published:** 2024-02-12

**Authors:** Derrick Lim, William Pei, Jae W. Lee, Kristin E. Musselman, Kei Masani

**Affiliations:** 1 Institute of Biomedical Engineering, University of Toronto, Toronto, ON Canada; 2 KITE - Toronto Rehabilitation Institute, University Health Network, Toronto, ON Canada; 3 Department of Physical Therapy, Temerty Faculty of Medicine, University of Toronto, Toronto, Canada; 4 Rehabilitation Science Institute, Temerty Faculty of Medicine, University of Toronto, Toronto, Canada

**Keywords:** Functional electrical stimulation, Standing balance, Visual feedback training, Depth camera

## Abstract

Individuals with incomplete spinal-cord injury/disease are at an increased risk of falling due to their impaired ability to maintain balance. Our research group has developed a closed-loop visual-feedback balance training (VFBT) system coupled with functional electrical stimulation (FES) for rehabilitation of standing balance (FES + VFBT system); however, clinical usage of this system is limited by the use of force plates, which are expensive and not easily accessible. This study aimed to investigate the feasibility of a more affordable and accessible sensor such as a depth camera or pressure mat in place of the force plate. Ten able-bodied participants (7 males, 3 females) performed three sets of four different standing balance exercises using the FES + VFBT system with the force plate. A depth camera and pressure mat collected centre of mass and centre of pressure data passively, respectively. The depth camera showed higher Pearson's correlation (*r* > 98) and lower root mean squared error (RMSE < 10 mm) than the pressure mat (*r* > 0.82; RMSE < 4.5 mm) when compared with the force plate overall. Stimulation based on the depth camera showed lower RMSE than that based on the pressure mat relative to the FES + VFBT system. The depth camera shows potential as a replacement sensor to the force plate for providing feedback to the FES + VFBT system.

## Introduction

Spinal cord injury/disease refers to damage to the spinal cord that results in sensory and/or motor impairment below the level of injury. In Canada, approximately 85, 000 people are living with spinal cord injury/disease with 11,000 new cases each year [1]. Spinal cord injury/disease can be classified as either complete or incomplete depending on whether sensory and motor functions below the injury are completely lost or partially retained. Individuals with motor incomplete spinal cord injury (iSCI) can regain their ability to walk; however, due to the effect of sensorimotor impairments on their standing posture, about 69–78% of individuals with iSCI experience falls at least once a year [2, 3]. Physical injuries from falls can result in reduced mobility and participation which can lead to non-physical consequences, such as dependence and reduced quality of life.

Currently, ambulatory inpatients with iSCI spend on average, a mere 2.0 ± 2.0 h on balance training over the course of their entire inpatient stay [4]. Conventional balance therapy focuses on increasing muscle strength and improving task-specific balance reactions [5–8]. In addition to these components, balance control also relies on sensory information from the somatosensory, visual, and vestibular systems. Vision is especially important for individuals with iSCI as studies have shown them to be more dependent than able-bodied individuals on visual cues for their standing balance [7, 9, 10]**.** As such, there has been research into incorporating visual feedback into balance rehabilitation exercises to provide a more targeted approach to balance therapy. Balance training in visual feedback balance training (VFBT) involves the participant shifting their body toward a target location provided on a screen along with the relative location of their body. Studies on VFBT have shown that it improves standing [7] and sitting [9] postural control in individuals with stroke and spinal cord injury. VFBT was shown to decrease root mean squared distance and mean velocity by 10–30 mm and 10–20 mm/s, respectively, in standing [7]. Similarly, VFBT helped to decrease the difference between optimal and actual movements in sitting [9], specifically, the movement duration, reaching error, directional error, extent error, and normalized jerk. In both papers, percent changes were not calculated for these measures and varied depending on the measures. Studies suggest that improvements in postural control due to VFBT can be attributed to sensorimotor integration and increased coordination through task-specific training [7, 9–13].

Two common measures for providing visual feedback of the participant’s body movement are body centre of mass (COM) [14–16] and centre of pressure (COP) [17–19]. The gold standard for capturing COM and COP is the motion capture system and the force plate, respectively. Between the two methods, COP from the force plate is easier to acquire; using the motion capture system requires setting up a network of cameras and data acquisition systems as well as a multistep preparation process of calibrating the system and placing markers on the participant. Studies using visual COP feedback have shown that it significantly improved standing balance in individuals with iSCI [7, 10]. In the study by Tamburella et al., the experimental group receiving VFBT improved clinical (i.e., Berg Balance Scale) and biomechanical (e.g., COP sway path, COP mean velocity, COP sway area, etc.) measures by 35–70% [10].

As ankle muscles play an important role in maintaining standing balance, activation of the weakened ankle muscles within populations such as individuals with iSCI may be a beneficial component of balance training [20]. Functional electrical stimulation (FES) is a method for artificially inducing contractions in paralyzed muscles by applying a high frequency current to targeted peripheral nerves using transcutaneous electrodes. This allows for individuals with limited motor function to participate in activities, such as standing [7, 21, 22], cycling [23–25], and stepping [26]. FES has been shown to yield general health benefits for individuals with iSCI, such as reducing muscle atrophy, increasing muscle mass, and improving blood circulation [27–30]. In addition, further studies have shown that using FES can facilitate neuroplasticity thus improving and restoring motor functions. For example, FES has been demonstrated in several studies to improve upper limb motor function by facilitating positive neural plasticity observed in the strengthening of corticospinal connections [31].

Some studies have also investigated the combination of visual feedback and FES for standing balance rehabilitation [18, 32]. Galeano et al. proposed an open-loop system for assessing and training balance using static posturography and FES using both the Nintendo Wii Balance Board and the Microsoft Kinect to provide visual feedback of the COP and body segment kinematics [18]; the study evaluated the system with six able-bodied participants presenting preliminary data only verifying the functionality of the system and not any orthotic or therapeutic effect. Audu et al. demonstrated the feasibility of using closed-loop FES to stabilize standing posture in two individuals with iSCI [32]. The system consisted of a force plate for COP feedback and implanted electrodes for FES during perturbation resistance training [32]. Both participants had received an implanted neural prosthesis that targeted their trunk, hip, knee, and ankle muscles. A major limitation of the system was its use of implanted electrodes, making the system invasive and unsuitable for therapeutic use.

Some studies have explored the use of alternative sensors to the force plate for feedback in rehabilitation systems [19, 26, 33–35]. Some common alternative commercial sensors are the depth camera and pressure mat which are relatively inexpensive, portable, and easy to setup. While there have been studies that investigate the use of depth cameras and pressure mats for feedback to FES systems [26, 33] and balance rehabilitation [19, 34, 35], to our knowledge, there has not been studies that validate a hybrid FES and VFBT system.

Previous studies by our research group have developed a novel VFBT system coupled with closed-loop FES using transcutaneous electrodes for rehabilitation of standing balance (FES + VFBT system) [13, 21]. The proposed FES + VFBT system capitalizes on FES-driven neural plasticity along with VFBT to improve lower limb motor function and standing balance. The system guides the user through a series of balance training exercises, where the participant controls a cursor using their COP as measured by a force plate. In the exercises, the participant moves their cursor toward target locations displayed on a screen. As the user shifts their COP in response to an exercise, FES is applied to their plantarflexor and/or dorsiflexor muscles to aid the user in completing the balance task. As part of the system, the FES controller mimics physiological activation of muscles during the balance tasks. A pilot study evaluating the training effect of this current FES + VFBT system with five individuals with iSCI yielded promising results showing increased range in COP displacement and improved clinical balance scores [13, 21]. However, translation of this system into the clinics is limited by the required equipment, particularly the force plate that is used for both the visual feedback and for the FES controller. Force plates are not easily accessible, require technical setup, and cost thousands of dollars. As such, the current iteration of the FES + VFBT system is not ready for clinical use.

Here we investigated the feasibility of using a depth camera and pressure mat for COM feedback to the FES + VFBT system. By replacing the force plate with a cheaper and more accessible sensor, the system will be more portable and affordable improving its overall accessibility for clinical use. We hypothesize that clinically accessible sensors such as the depth camera and pressure mat are feasible replacements to the force plate.

## Results

### Comparison of approximated COM displacement using depth camera and pressure mat with cop from force plate

**Fig. 1:**
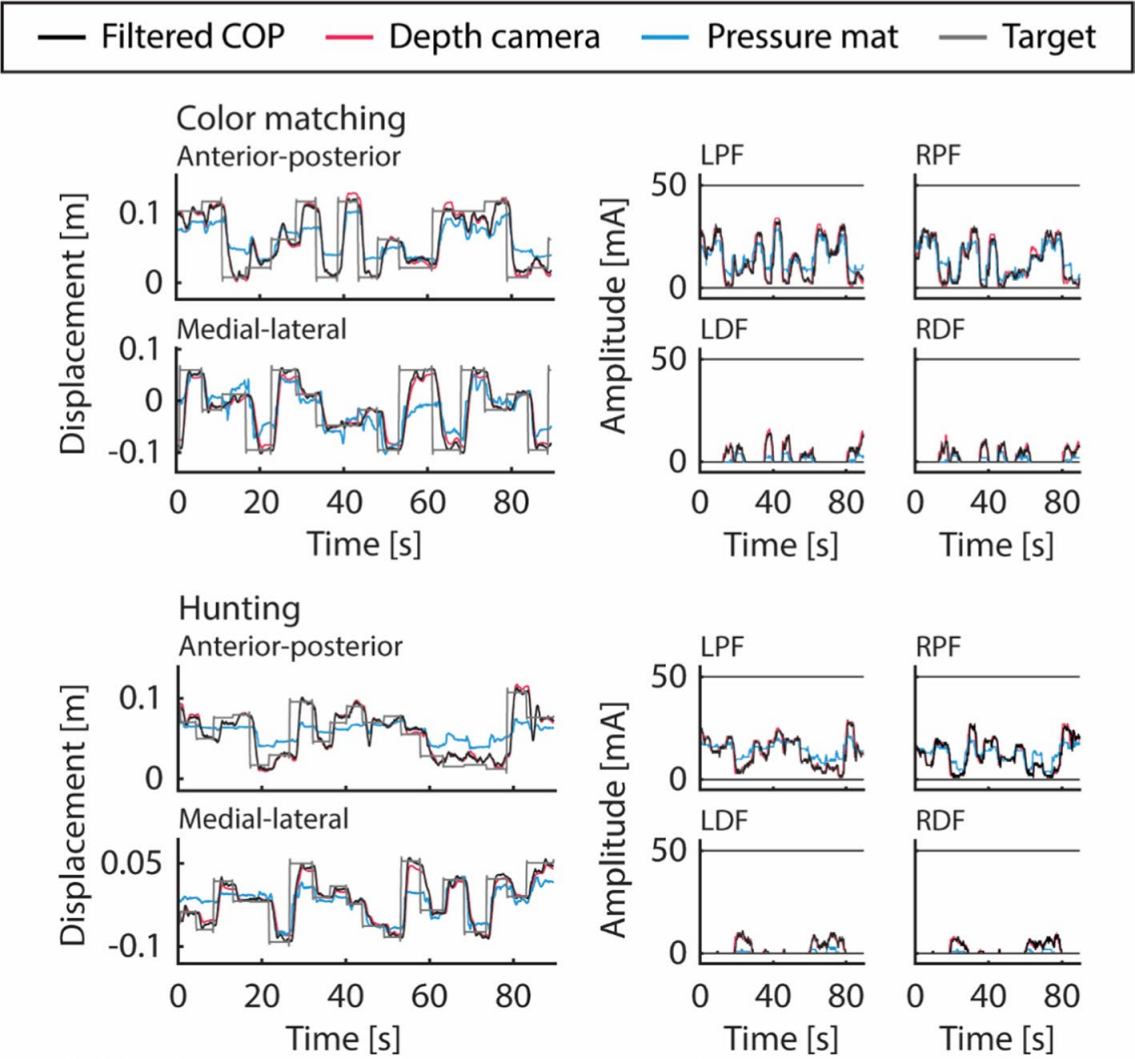
Filtered COP and COM time series (left) and stimulation output (right) for color matching and hunting VFBT exercises using different inputs: filtered COP (black), COM from the Kinect v2 depth camera (red), and COP from the pressure mat (blue). The horizontal lines (dark grey) on the stimulation output (right) represent the participant’s motor threshold and maximum tolerable stimulation intensity. Stimulation outputs are shown for the left and right plantarflexors (LPF and RPF) as well as the left and right dorsiflexors (LDF and RDF)

**Fig. 2:**
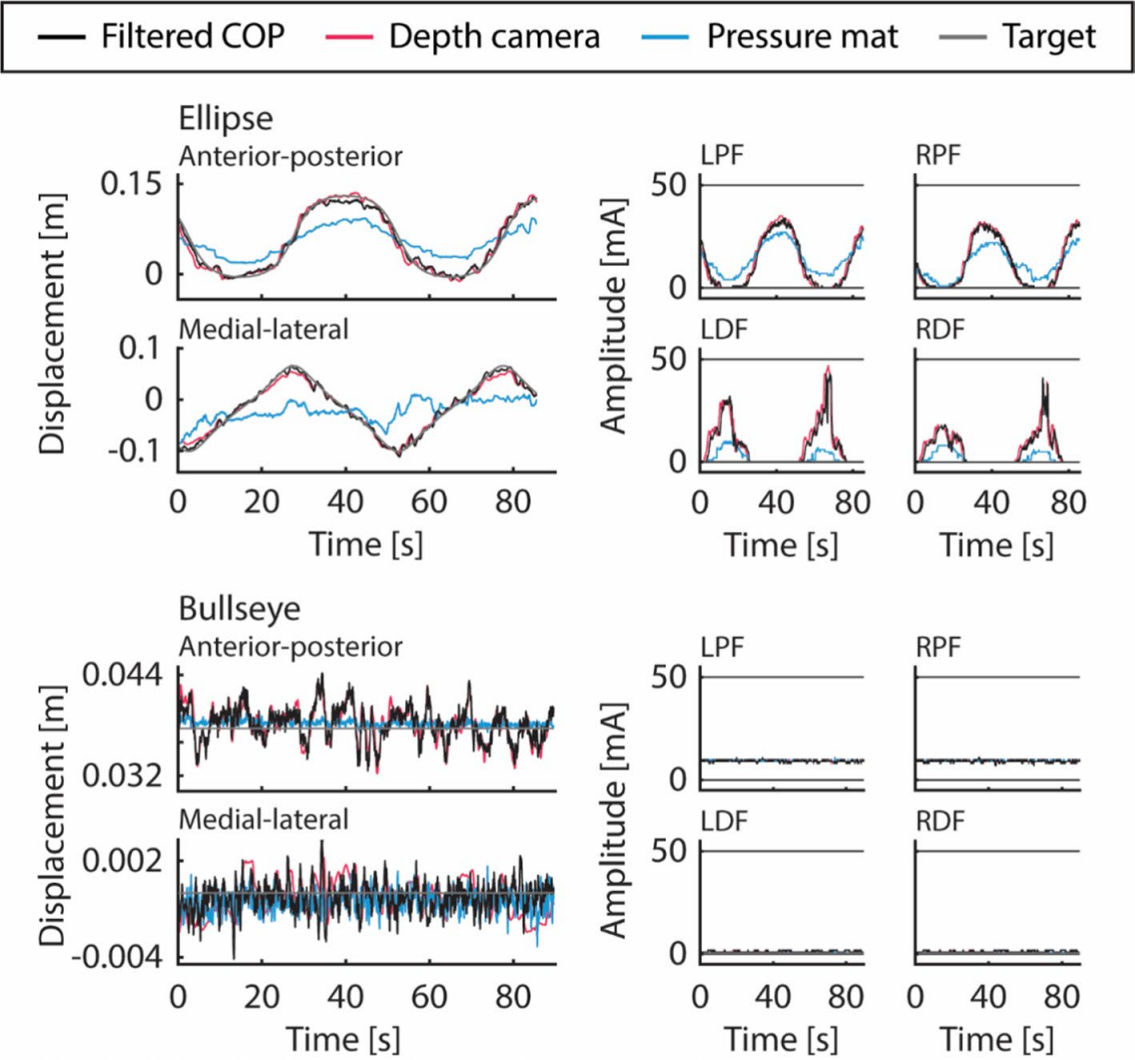
Filtered COP and COM time series (left) and stimulation output (right) for ellipse and bullseye VFBT exercises using different inputs: filtered COP (black), COM from the Kinect v2 depth camera (red), and COP from the pressure mat (blue). The horizontal lines (dark grey) on the stimulation output (right) represent the participant’s motor threshold and maximum tolerable stimulation intensity. Stimulation outputs are shown for the left and right plantarflexors (LPF and RPF) as well as the left and right dorsiflexors (LDF and RDF)

Figures 1, 2 show the typical COM approximation time series during the VFBT exercises and the corresponding stimulation using: (1) the force plate (black) as part of the current FES + VFBT system, (2) depth camera (red), and (3) the pressure mat (blue).

**Fig. 3:**
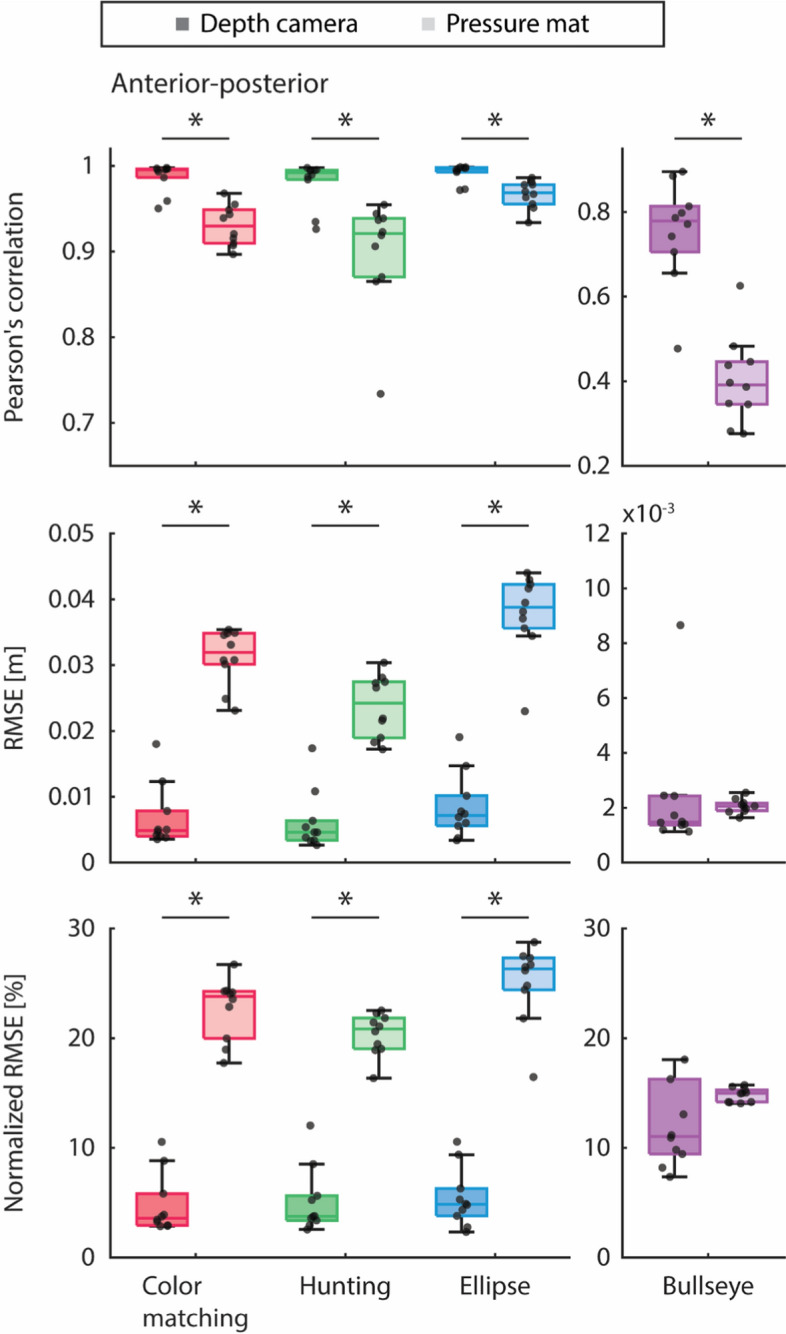
In the anterior–posterior direction, Pearson’s correlation, root mean squared error (RMSE), and normalized RMSE of the Kinect v2 depth camera (shown in darker color) and the pressure mat (shown in lighter color) compared against the force plate, separated by the type of VFBT exercise (i.e., color matching, hunting, ellipse, and bullseye shown in red, blue, green, and purple, respectively). Significant difference (*p* < 0.05) between sensors is indicated by *

**Fig. 4:**
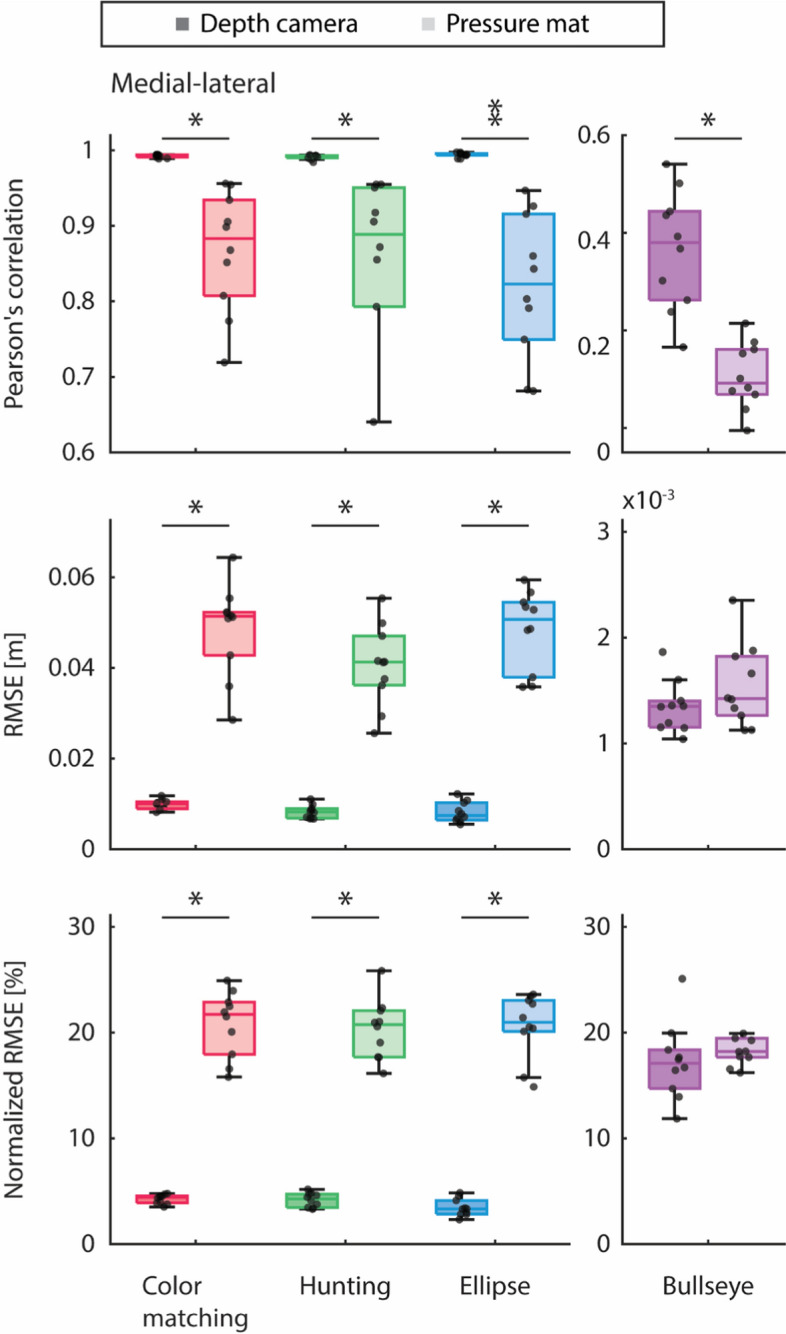
In the medial–lateral direction, Pearson’s correlation, root mean squared error (RMSE), and normalized RMSE of the Kinect v2 depth camera (shown in darker color) and the pressure mat (shown in lighter color) compared against the force plate, separated by the type of VFBT exercise (i.e., color matching, hunting, ellipse, and bullseye shown in red, blue, green, and purple, respectively). Significant difference (*p* < 0.05) between sensors is indicated by *

Figure 3, 4 and Tables 1, 2, 3 show and present the sample distribution of the Pearson’s correlation, root mean squared error (RMSE), and normalized RMSE between the depth camera’s COM with the force plate’s filtered COP and the pressure mat’s COP correlation with the force plate’s filtered COP for all VFBT exercises in both anterior–posterior and medial–lateral directions.

**Table 1:** Pearson’s correlation of the force plate’s filtered COP with the depth camera’s COM and the pressure mat’s COP across the VFBT balance exercises for the anterior–posterior (AP) and medial–lateral (ML) direction

	Depth camera		Pressure mat	
	AP (Mean ± SD)	ML (Mean ± SD)	AP (Mean ± SD)	ML (Mean ± SD)
Color	0.99 ± 0.02	0.99 ± 0.00	0.93 ± 0.02*	0.87 ± 0.08*
Hunt	0.98 ± 0.03	0.99 ± 0.00	0.90 ± 0.07	0.83 ± 0.15
Ellipse	0.99 ± 0.01	0.99 ± 0.00	0.97 ± 0.02*	0.82 ± 0.10*
Bullseye	0.75 ± 0.12*	0.37 ± 0.12*	0.40 ± 0.10*	0.11 ± 0.07*

*Indicates statistical difference (*p* < 0.05) between the Pearson’s correlation in the AP and ML direction

**Table 2:** Root mean squared error of the force plate’s filtered COP with the depth camera’s COM and the pressure mat’s COP across the VFBT balance exercises for the anterior–posterior (AP) and medial–lateral (ML) direction

	Depth camera		Pressure mat	
	AP (Mean ± SD m)	ML (Mean ± SD m)	AP (Mean ± SD m)	ML (Mean ± SD m)
Color	0.007 ± 0.005	0.010 ± 0.001	0.031 ± 0.004*	0.049 ± 0.010*
Hunt	0.006 ± 0.005*	0.008 ± 0.001*	0.024 ± 0.005*	0.041 ± 0.008*
Ellipse	0.008 ± 0.005	0.008 ± 0.002	0.038 ± 0.006*	0.048 ± 0.009*
Bullseye	0.002 ± 0.002	0.001 ± 0.000	0.002 ± 0.000*	0.002 ± 0.000*

*Indicates statistical difference (*p* < 0.05) between the RMSE in the AP and ML direction

**Table 3:** Normalized RMSE of the force plate’s filtered COP with the depth camera’s COM and the pressure mat’s COP across the VFBT balance exercises for the anterior–posterior (AP) and medial–lateral (ML) direction

	Depth camera		Pressure mat	
	AP (Mean ± SD %)	ML (Mean ± SD %)	AP (Mean ± SD %)	ML (Mean ± SD %)
Color	4.82 ± 2.74	4.27 ± 0.44	22.67 ± 2.83	20.81 ± 3.11
Hunt	5.14 ± 2.98	4.18 ± 0.67	20.35 ± 1.91	20.34 ± 2.82
Ellipse	5.45 ± 2.66*	3.45 ± 0.82*	25.04 ± 3.59*	20.59 ± 3.07*
Bullseye	17.10 ± 17.77*	17.22 ± 3.62*	14.81 ± 0.63*	19.41 ± 4.18*

*Indicates statistical difference (*p* < 0.05) between the normalized RMSE in the AP and ML direction

Friedman’s test showed statistically significant difference (*p* < 0.05) between the depth camera’s COM correlation with the force plate’s filtered COP and the pressure mat’s COP correlation with the force plate’s filtered COP for all VFBT exercises. For RMSE and normalized RMSE between the depth camera’s COM and pressure mat’s COP with the force plate’s filtered COP, statistically significant differences were found between the results of the two sensors for all the dynamic VFBT exercises (i.e., color matching, hunting, and ellipse). The same statistical trends are evident in both the anterior–posterior and medial–lateral directions. Friedman’s test was also applied to differences between the anterior–posterior and medial–lateral directions within each sensor (i.e., depth camera and pressure mat) for each outcome measure (i.e., Pearson’s correlation, RMSE, and normalized RMSE).

### Comparison of stimulation based on approximated COM displacement using depth camera and pressure mat against that based on COP from force plate

**Fig. 5:**
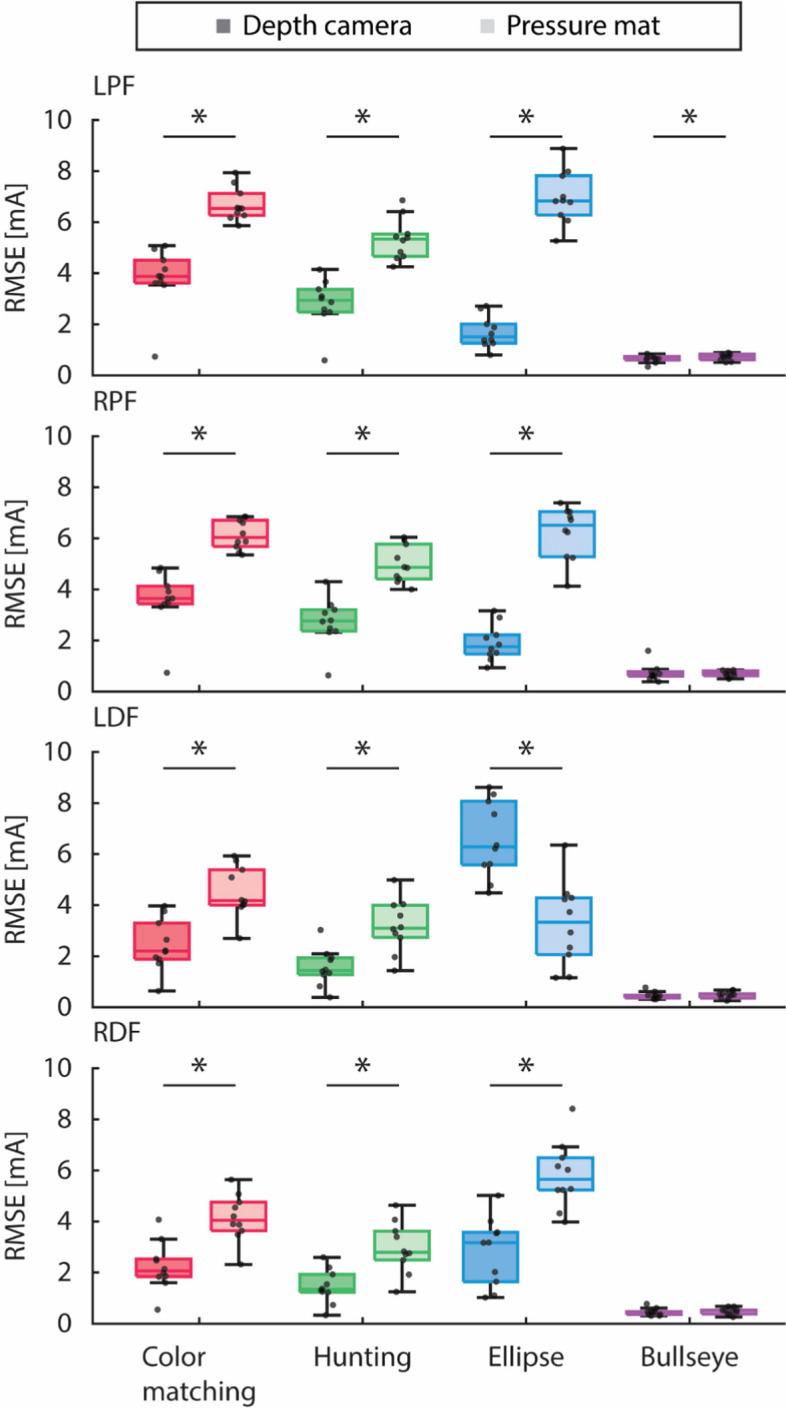
Root mean squared error of the stimulation based on the Kinect v2 depth camera’s COM (shown in darker color) and the pressure mat’s COP (shown in lighter color) compared against that based on the force plate’s filtered COP, separated by the type of VFBT exercise (i.e., color matching, hunting, ellipse, and bullseye shown in red, blue, green, and purple, respectively). Results are shown for the left and right plantarflexors (LPF and RPF) as well as the left and right dorsiflexors (LDF and RDF). Significant difference (*p* < 0.05) between sensors is indicated by *

Figure 5 and Tables 4, 5 show and present the RMSE of the stimulation based on the depth camera’s COM (shown in darker color) and the pressure mat’s COP (shown in lighter color) compared against that based on the force plate’s filtered COP, separated by the of VFBT exercise.

**Table 4:** Root mean squared error of the stimulation based on the Kinect v2’s COM and the pressure mat’s COP compared against that based on the force plate’s filtered COP across the VFT balance exercises for the plantarflexors

	Left plantarflexors (LPF)		Right plantarflexors (RPF)	
	Depth camera (Mean ± SD mA)	Pressure mat (Mean ± SD mA)	Depth camera (Mean ± SD mA)	Pressure mat (Mean ± SD mA)
Color	3.11 ± 0.99	3.32 ± 1.19	3.45 ± 1.02	2.16 ± 1.49
Hunt	2.25 ± 0.70	2.57 ± 1.21	2.30 ± 0.95	1.44 ± 0.96
Ellipse	1.81 ± 1.04	3.58 ± 1.36	1.98 ± 0.52	2.32 ± 1.37
Bullseye	0.54 ± 0.18	0.78 ± 0.45	0.62 ± 0.16	0.43 ± 0.13

**Table 5:** Root mean squared error of the stimulation based on the Kinect v2’s COM and the pressure mat’s COP compared against that based on the force plate’s filtered COP across the VFT balance exercises for the dorsiflexors

	Left dorsiflexors (LDF)		Right dorsiflexors (RDF)	
	Depth camera (Mean ± SD mA)	Pressure mat (Mean ± SD mA)	Depth camera (Mean ± SD mA)	Pressure mat (Mean ± SD mA)
Color	5.75 ± 1.50	5.26 ± 1.60	5.61 ± 1.24	4.88 ± 0.93
Hunt	4.72 ± 1.33	4.16 ± 1.67	4.09 ± 1.33	3.51 ± 1.03
Ellipse	6.54 ± 1.29	7.30 ± 1.12	5.82 ± 1.18	5.90 ± 1.01
Bullseye	0.57 ± 0.19	0.57 ± 0.09	0.70 ± 0.19	0.55 ± 0.17

Friedman’s test showed statistically significant difference (*p* < 0.05) between the stimulation intensities based on the depth camera’s COM and that based on the pressure mat’s COP for all muscle groups (i.e., left and right plantarflexors and dorsiflexors) during dynamic VFBT exercises (i.e., color matching, hunting, and ellipse). During the bullseye exercise, only the left plantarflexor showed statistically significant difference between stimulation intensities based on the depth camera’s COM and that based on the pressure mat’s COP.

## Discussion

### Comparison of approximated COM displacement using depth camera and pressure mat with COP from force plate

COM from the depth camera was significantly more correlated and had lower RMSE with filtered COP from the force plate than COP from the pressure mat. This trend was observed in both the anterior–posterior and the medial–lateral directions. Looking at the time series from the depth camera, pressure mat, and force plate, the amplitude of the pressure mat’s COP was often much smaller as can be observed qualitatively in Figs. 1, and 2 and quantified by RMSE of ~ 0.03–0.04 m for dynamic exercises and < 0.001 m for the bullseye exercise in Figs. 3, and 4. Thus, even when the pressure mat’s COP followed the trend of the filtered COP from the force plate, its RMSE was much greater than that of the depth camera. One possible factor for this was the pressure mat’s inability to measure horizontal forces. During leaning, the force exerted by the body through the feet onto the pressure mat consisted of both vertical and horizontal components. Based on the equation for calculating COP in section “Data processing”, disregarding the horizontal force component will affect the overall COP.

For the depth camera, the Pearson’s correlation in the anterior–posterior direction was significantly higher than that in the medial–lateral direction during the bullseye exercise. One possible implication for this was that even when the body detection algorithm does not accurately track the participant’s movement in the medial–lateral direction, tracking of the participant’s movement in the anterior–posterior direction was not negatively affected. Few significant differences between the directions were seen in the depth camera’s RMSE results. One implication of this was that the depth camera’s body detection for the COM time series in the medial–lateral direction was good enough that it did not impact the COM time series in the anterior–posterior direction. One concern was that if the body detection was not sufficiently accurate, an object in the background might be mislabeled as one of the participant’s body joints. This would significantly affect the calculation for the whole-body COM; as this is not the case, the body detection algorithm was not a limiting factor for the depth camera. Significant differences between the directions in the depth camera’s normalized RMSE results were observed in the ellipse and bullseye exercises. These were two of the slower movement tasks in the four VFBT exercises. This suggested that the depth camera captured a smaller percentage of the amplitude of the participant’s movements in slower moving tasks.

Significant differences between the directions in the pressure mat’s Pearson correlation results suggested that the pressure mat was better at tracking the participant’s whole-body COM in the anterior–posterior direction than in the medial–lateral direction. Similar significant differences between the directions in the pressure mat’s RMSE results supported the implication that the whole-body COM was better captured in the anterior–posterior direction. Like the depth camera, significant differences between the directions were found for the ellipse and bullseye exercises for the pressure mat’s normalized RMSE results. The same inference can be made that for slower moving tasks the pressure mat captured a smaller percentage of the amplitude of the participant’s movements.

### Comparison of stimulation based on approximated COM displacement using depth camera and pressure mat against that based on COP from force plate

Apart from the bullseye exercise, the stimulation based on the depth camera has significantly lower RMSE than that based on the pressure mat when compared against the stimulation from the current FES + VFBT system using the force plate. For the Kinect, RMSE of the stimulation intensities for the bullseye exercise and for the dynamic VFBT exercises were 0.6 mA and 4 ± 2 mA, respectively; for the pressure mat, the RMSE of the stimulation intensities for the bullseye exercise and for the dynamic VFBT exercises were also 0.6 mA and 4 ± 3 mA, respectively. During bullseye exercise, the stimulation intensities were around 10 mA; as such, RMSE of 0.6 mA is very little compared to what the participant experiences. Likewise, during the dynamic VFBT exercises, the stimulations intensities go up to 90% of the participant’s maximum tolerable intensity which can reach 60 mA; RMSE of 4 mA would also be a small percentage of the stimulation applied to the participant.

### Limitations

One future step for the study is to test the source of the errors for the depth camera (i.e., Kinect v2) and pressure mat. For the Kinect v2, a potential factor to investigate is its setup. In general, the Kinect v2 was placed, such that it faced the frontal plane of the user; however, there were two parameters that can be changed: its height and its viewing angle. In this study, the Kinect v2 was placed 1.67 m from the ground and at a 20° angle. This allowed for optimal view of the participants in the current setup. The Python library used to interface with the depth camera does not have a function to consider these two parameters to calibrate the Kinect v2’s depth calculations. This means that a potential source of error by the Kinect v2 could be corrected by taking these parameters into consideration.

For the pressure mat, a potential factor for its error is its inability to capture horizontal forces. A next step may be to use just the vertical component of the force plate to calculate COP and see if that matches the decreased amplitude of the pressure mat’s COP. In addition, the sensors in the pressure mat are placed in a 15 × 30 grid with 1 cm in between each of the sensor (see section "Depth camera and pressure mat"). By increasing the density of the sensors in the pressure mat, we might be able to also improve its spatial resolution. While the pressure mat operated at a lower frequency than the depth camera and FES + VFBT system, its sampling frequency is not identified as a limitation as COM displacement is expected to be well-captured in the low frequency range. Bad time resolution due to low sampling frequency would result in abrupt changes in the COP displacement time series from the pressure; however, this was not observed.

Finally, while the study has used able-bodied individuals for evaluating the feasibility of these alternatives sensors for providing COM feedback to FES + VFBT system, the final system will be used with individuals with iSCI. Two major expected differences between these two populations are poorer balance capabilities and larger range in motor threshold and tolerable stimulation intensities. Poorer balance will likely result in smaller ranges in COM displacement. This may affect result in slightly lower correlation results, which depend on the variance of the time series. However, the trends between the pressure mat and depth camera relative to the force plate will remain the same. Absolute and normalized errors should remain very similar as they are independent of how the movement of the subject—meaning that the corresponding stimulation should have the same range of errors. In that case with larger range in stimulation intensities, the proportionate stimulation intensity errors will also be smaller.

## Conclusion

The depth camera’s COM showed higher correlation and lower RMSE than the pressure mat’s COP with the force plate’s filtered COP. Furthermore, analysis of the stimulation based on these inputs showed that stimulation using the depth camera showed lower RMSE than that using the pressure mat compared against that using the force plate. As such, between the two sensors, the depth camera showed much more potential as a low-cost, portable sensor than the pressure mat and is a feasible option as a replacement to the force plate for use in the FES + VFBT system. Results from this study has broad implications not just for alternate sensors to the force plate but also for the development of a more clinically accessible FES + VFBT system. While the FES + VFBT system was initially developed to target individuals with iSCI, it may be applicable to other populations with non-progressive, upper motor neuron damage resulting in balance impairments, such as adults living with stroke.

## Materials and methods

### Participants

A total of 10 able-bodied participants (7 male, 3 female) aged 25.3 ± 4.7 years (mean ± SD), with an average height of 174.0 ± 6.4 cm and weight of 71.3 ± 15.4 kg, with no history of neurological disorders, participated in this study. This study was approved by the Research Ethics Board of the University Health Network in accordance with the Declaration of Helsinki on the use of human participants in experiments.

### Study protocol

**Fig. 6:**
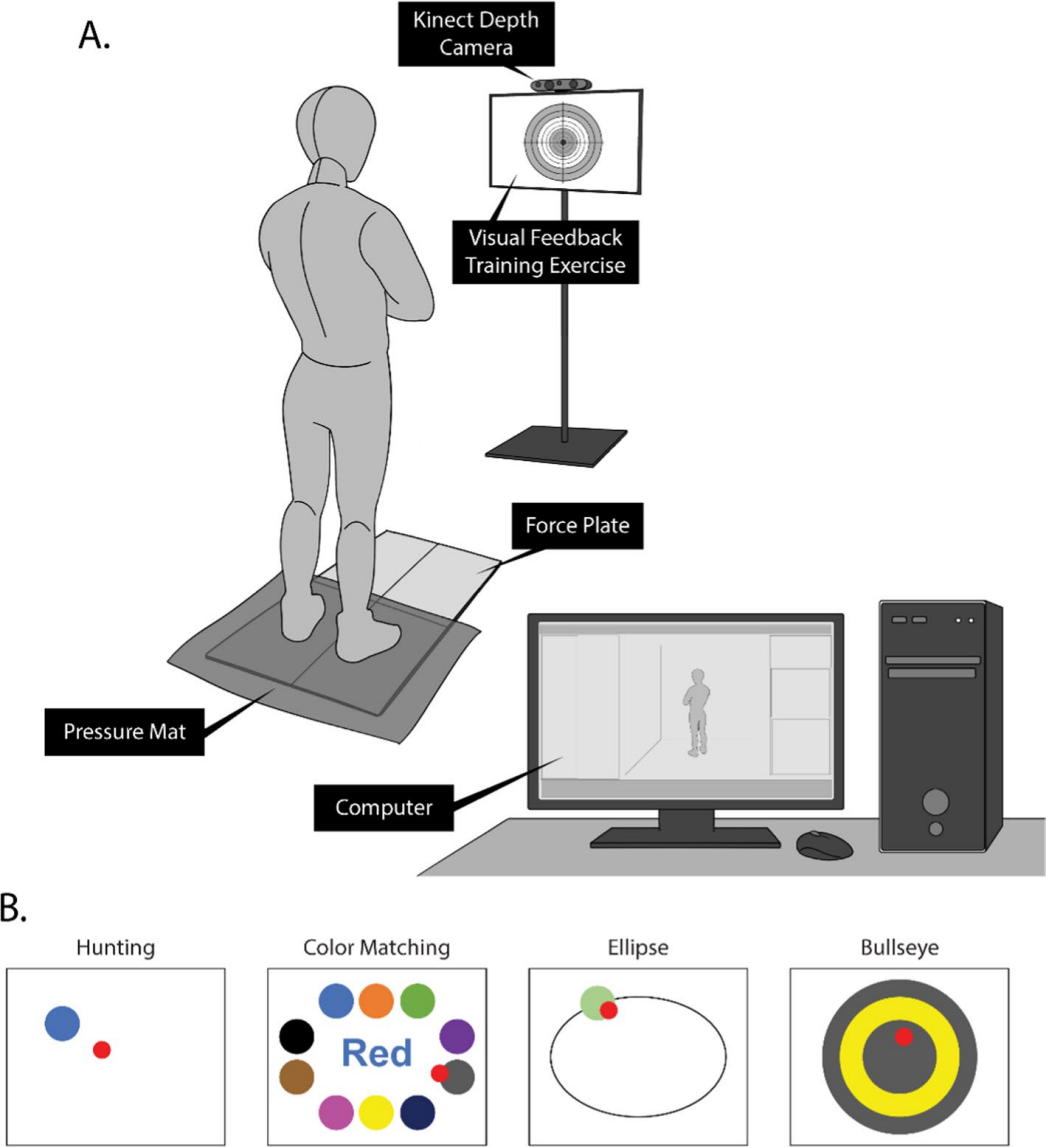
**A** Schematic of the experimental setup with the participant playing a visual feedback balance exercise, while Kinect v2 depth camera and pressure mat information are simultaneously collected. **B** Schematic of visual feedback training exercises used in the current FES + VFBT system [11]. The red cursor represents the participant’s COP. The participants played through three sets of four VFBT exercises. Within each set, the order of the exercises (e.g., hunting, color matching, etc.) were randomized

The participants were asked to complete three sets of four 100 s VFBT exercises using the FES + VFBT system (see Fig. 6). There are four variations of VFBT exercises: (1) hunting, (2) color matching, (3) ellipse, and (4) bullseye exercises. In each balance exercise, the participant’s COP was represented by a red cursor. In Hunting, a new target is generated at a random location on the screen after the participant stayed within the target for 3 s. In Color Matching, different colored targets propagate the edge of the screen. A text prompt was shown in the middle of the screen, and the participant was tasked to move their cursor to the color of the text prompt (e.g., if the prompt is “Red” but the font color is blue, then the correct target color is blue). In Ellipse, the target moves in a constant elliptical trajectory and the participant was tasked with following the target as closely as possible. In Bullseye, the participant was tasked with staying in the center of the screen as much as they can. In all the exercises, the participant was asked to keep their arms crossed over their chest and to have their body as straight as possible, leaning predominantly only using their ankles.

### FES + VFBT system

In the current FES + VFBT system, force plate information is used for both visual feedback for the balance exercises as well as input to the proportionate-derivative (PD) controller to calculate the appropriate stimulation for the soleus and tibialis anterior. The current FES + VFBT system runs at 20 Hz and COP, target, and stimulation data for the balance exercises are saved in log files.

The FES + VFBT system uses the force plate (AccuSway-Dual, Advanced Mechanical Technology Inc., Watertown, USA) for estimated COM feedback. The force plate is interfaced with both the computer running the FES + VFBT system as well as a computer running Cortex 3.1 (Motion Analysis Corp., Rohnert Park, CA) that saved the force plate’s raw values using a DAQ device. Raw data from the force plate was recorded at 2000 Hz.

### Depth camera and pressure mat

The Kinect v2 (Microsoft, USA) depth camera was placed directly in front facing the participant. A custom Python script using the Pykinect package was used to visualize and collect 3D body joint displacement data. Body detection by the Kinect v2 was used for the medial–lateral displacement time series of the joints, while the depth stream of the camera was used to approximate the anterior–posterior position of the joints. The program sampled at nonuniform rates ranging from 30 to 100 Hz. Each trial was individually recorded.

The pressure mat (Myant, Canada) was placed directly over the force plates with its bottom edge aligned with that of force plates. The current iteration of the pressure mat consisted of a matrix of 15 × 30 sensors, each measuring 1.8 cm by 1.0 cm, evenly spaced with 1.0 cm between each sensor. The pressure mat was designed to sample at around 11 Hz and has its own user interface for adjusting gain and data cutoff values, which were set to 2 and 0 to 555, respectively. Each trial was individually recorded.

### FES + VFBT simulation

A custom written program was used to simulate the PD controller built in the FES + VFBT system. As COP and COM data from the depth camera and pressure mat were collected independently from the FES + VFBT system, this simulation is necessary for comparison of the stimulation from the FES + VFBT system with stimulation from the system if it were to use inputs from the depth camera or pressure mat in real time. Simulation of the stimulation, which ranged from 0 to 50 mA (maximum tolerable stimulation intensity), to the left and right dorsiflexors and plantarflexors were calculated for all trials. The stimulation range reflected potential values of stimulation experienced by the participants when using the FES + VFBT system.

### Data processing

COP data from the current FES + VFBT system were pre-filtered by the system using a moving average filter with a window length of three data points. Target location information from the current FES + VFBT system was filtered using a zero-phase fourth-order lowpass Butterworth filter with a cutoff frequency of 10 Hz [10, 11].

Force and COP data from the force plate were filtered using a zero-phase fourth-order lowpass Butterworth filter with a cutoff frequency of 10 Hz and then downsampled to 20 Hz. Global COP from the bilateral force plates was calculated using the formula below and then filtered using zero-phase fourth-order lowpass Butterworth filter with cutoff frequency of 0.4615 Hz:

1 $${{\text{COP}}}_{x}=\frac{{F}_{r,z}* {{\text{COP}}}_{r,x}+{F}_{l,z}* {{\text{COP}}}_{l,x}}{{F}_{r,z}+{F}_{l,z}}$$

where ${F}_{r,z}$ and ${F}_{l,z}$ are the vertical forces measured from the right and left force plates; similarly, ${{\text{COP}}}_{r,x}$ and ${{\text{COP}}}_{l,x}$ refer to the local COP measured from the right and left force plates.

Joint displacement data from the depth camera were first resampled to a constant frequency of 20 Hz and then filtered using a zero-phase fourth-order lowpass Butterworth filter with a cutoff frequency of 5 Hz [36, 37]. COM from the depth camera was calculated using Winter’s anthropometric data [38] based on a 5-link model consisting of the average of the left and right shank and thigh and the head, arms, and trunk (HAT), bounded by the greater trochanter and the glenohumeral joint. The shank was calculated from the ankle (i.e., lateral malleolus) and knee (i.e., femoral condyles), while the thigh was calculated using the knee and hip (i.e., greater trochanter).

COP from the pressure mat was calculated using the following equations:

2 $${{\text{COP}}}_{x}=\frac{\left({\sum }_{x=1}^{30}p*x\right)*0.1}{\left({\sum }_{x=1}^{30}{\sum }_{y=1}^{15}p\right)*2}$$

3 $${{\text{COP}}}_{y}=\frac{\left({\sum }_{y=1}^{15}p*y\right)*0.1}{\left({\sum }_{x=1}^{30}{\sum }_{y=1}^{15}p\right)*2}$$

where *p* is the pressure recorded by each individual sensing cell in the pressure mat, 0.1 m is the distance between the cells, and 2 is the gain applied to the raw data during data collection. Data from the pressure mat were resampled to match a 20 Hz sampling rate.

To synchronize the force plate data, depth camera data, and the pressure mat data, the cross-correlation function was used to determine the delays between the systems associated with the highest correlation. The delays were then verified visually and used to time-sync the signals.

COM time series as captured by the alternative sensors were compared against that estimated from the force plate; this comparison was evaluated through Pearson’s correlation as well as absolute and normalized RMSE. Normalized RMSE was calculated by dividing the RMSE by the dynamic range of the force plate’s filtered COP time series. For each participant, Pearson’s correlation, RMSE, and normalized RMSE were calculated for each of the 12 trials and then group averaged according to exercise type (e.g., hunting). This was calculated for both the depth camera and the pressure mat.

Absolute RMSE between stimulation intensities determined from the alternative sensors and the force plate were calculated and group averaged according to exercise type. This was also calculated for both the depth camera and the pressure mat. Pearson’s correlation and normalized RMSE were not calculated due to cases when stimulation time series are entirely 0 mA, which makes correlation or normalization difficult.

All of processing and analysis of the data were performed in MATLAB (R2021a).

### Statistical analysis

The Shapiro–Wilk test was used to check for the data’s normality. Friedman’s test was used to examine the effect of the alternate sensors (i.e., depth camera or pressure mat) on the correlation, RMSE, and normalized RMSE of the COP or COM, respectively, compared with COP from the force plate. This was performed for both the anterior–posterior and medial–lateral directions. Another Friedman’s test was used to examine the effect of the alternate sensors on RMSE of the stimulation compared with that from the FES + VFBT system. The statistical tests were performed in MATLAB (R2021a) using *p* < 0.05 as the significance level.

## Data Availability

The data sets used and analysed during the current study are available from the corresponding author on reasonable request.
